# Utilization of Integrated Child Development Services in India: Programmatic Insights from National Family Health Survey, 2016

**DOI:** 10.3390/ijerph17093197

**Published:** 2020-05-04

**Authors:** Sunil Rajpal, William Joe, Malavika A. Subramanyam, Rajan Sankar, Smriti Sharma, Alok Kumar, Rockli Kim, S. V. Subramanian

**Affiliations:** 1 Institute of Health Management Research, IIHMR University, Jaipur 302029, India; sunilrajpal27@gmail.com; 2 Institute of Economic Growth, Delhi 110007, India; william@iegindia.org; 3 Indian Institute of Technology, Gandhinagar 382355, India; malavika@iitgn.ac.in; 4 Tata Trusts, Mumbai 400005, India; rsankar@tatatrusts.org (R.S.); ssharma@tatatrusts.org (S.S.); 5 NITI Aayog, Government of India, New Delhi 110001, India; alokkumar.up@ias.nic.in; 6 Division of Health Policy and Management, College of Health Sciences, Korea University, Seoul 02841, Korea; rocklikim@korea.ac.kr; 7 Department of Public Health Sciences, Graduate School, Korea University, Seoul 02841, Korea; 8 Harvard Center for Population and Development Studies, Cambridge, MA 02138, USA; 9 Department of Social and Behavioral Sciences, Harvard T.H. Chan School of Public Health, Boston, MA 02115, USA

**Keywords:** ICDS, child undernutrition, childcare, India

## Abstract

The Integrated Child Development Services (ICDS) program launched in India in 1975 is one of the world’s largest flagship programs that aims to improve early childhood care and development via a range of healthcare, nutrition and early education services. The key to success of ICDS is in finding solutions to the historical challenges of geographic and socioeconomic inequalities in access to various services under this umbrella scheme. Using birth history data from the National Family Health Survey (Demographic and Health Survey), 2015–2016, this study presents (a) socioeconomic patterning in service uptake across rural and urban India, and (b) continuum in service utilization at three points (i.e., by mothers during pregnancy, by mothers while breastfeeding and by children aged 0–72 months) in India. We used an intersectional approach and ran a series multilevel logistic regression (random effects) models to understand patterning in utilization among mothers across socioeconomic groups. We also computed the area under the receiver operating characteristic curve (ROC-AUC) based on a logistic regression model to examine concordance between service utilization across three different points. The service utilization (any service) by mothers during pregnancy was about 20 percentage points higher for rural areas (60.5 percent; 95% CI: 60.3; 30.7) than urban areas (38.8 percent; 95% CI: 38.4; 39.1). We also found a lower uptake of services related to health and nutrition education during pregnancy (41.9 percent in rural) and early childcare (preschool) (42.4 percent). One in every two mother–child pairs did not avail any benefits from ICDS in urban areas. Estimates from random effects model revealed higher odds of utilization among schedule caste mothers from middle-class households in rural households. AUC estimates suggested a high concordance between service utilization by mothers and their children (AUC: 0.79 in rural; 0.84 in urban) implying a higher likelihood of continuum if service utilization commences at pregnancy.

## 1. Introduction

The Integrated Child Development Services (ICDS) is one of the world’s largest flagship programs with the primary aim of improving early childhood care and development [1,2,3,4,5]. The program was launched by the Government of India in 1975 and has adopted an integrated approach to deliver a range of healthcare, nutrition and early education services (broadly classified as supplementary nutrition, pre-school and non-formal education, health and nutrition education (HNE from now), immunization, health checkups and other referral services) for pregnant women, breastfeeding mothers and children up to six years. These services are delivered via Anganwadi Centers (AWCs) (An Anganwadi center provides basic health care in Indian villages. It is a part of the Indian public healthcare system. Basic healthcare activities include contraceptive counselling and supply, nutrition education and supplementation, as well as pre-school activities. (Source: https://data.gov.in/dataset-group-name/anganwadi-centers)) located at the village level and managed by grassroot level functionaries (Anganwadi Workers, AWWs). With the support from international organizations like World Bank (for ISSNIP (ISSNIP—ICDS Systems Strengthening and Nutrition Development Project (formerly called as ICDS-IV), Ministry of Child and Women Development, Government of India.)) and UNICEF, the program coverage has increased enormously from 4891 AWCs in 1975 to about 1.3 million currently operating AWCs in India [1].

According to recent National Family Health Survey (NFHS) 2015–16, every second child (0–59 months) in India suffers from some kind of nutritional failure (stunting, wasting and/or underweight) [6]. The problem intensifies manifold given the huge child population (i.e., 121 million in 2015) in India, which accounts for 18 percent of the global under-five population. The ICDS program is instrumental for improving the continuum of care and for addressing the high burden of maternal and child undernutrition in India. Nutritional well-being is one of the most crucial and effective pathways for human development, poverty reductions and economic development [7]. With the launch of the National Nutrition Mission in 2017 (now POSHAN (Prime Minister’s Overarching Scheme for Holistic Nourishment) *Abhiyaan*), there has been a greater focus on improving programmatic performance and inter-departmental coordination to accelerate reductions in child undernutrition [7]. These strategies are critical to achieve the POSHAN target of two percentage points per annum reductions in child stunting and underweight. ICDS is an important platform to deliver some of the most direct interventions—decentralized at village and AWCs level—designed under POSHAN *Abhiyaan* and therefore a thorough understanding on its utilization pattern can offer valuable insights regarding its contribution in the form of effective coverage and impact on nutritional status.

The key to success of ICDS is in finding solutions to the historical challenges of geographic and socioeconomic inequalities in access to various services under this umbrella scheme [8,9]. For instance, given the social residential segregation, geographical placement of AWCs under ICDS is one of the crucial factors that determine the utilization pattern across socioeconomic groups. Although previous studies have observed little programmatic impact of ICDS benefits on child’s nutritional status [10], given its universal design, accompanied by better coordination of multisectoral synergies under POSHAN *Abhiyaan*, ICDS can potentially bridge these equity gaps in health and nutrition outcomes among children. However, this demands a holistic understanding of the utilization patterns of various services offered under ICDS across rural and urban settings, as well as across broad demographic and socioeconomic groups of beneficiaries (mother and child).

The first objective of this study was to unravel intersectional inequalities in the utilization of key ICDS services among mothers (during pregnancy and breastfeeding) and children (below six years) in India using the recent wave of National Family Health Survey, 2015–16. While previous studies—in part—have assessed the utilization patterns and impact evaluation of ICDS using previous rounds of NFHS [11,12,13,14,15,16], most of these studies have found little impact of ICDS on child undernutrition, except the study by Kandpal (2011). A few studies based on regional-level data have also assessed the coverage and impact of ICDS on child undernutrition [17,18]. However, none of the studies have adopted an intersectional approach to identify the gaps in the utilization of services across socially and economically marginalized and disadvantaged groups. The intersectionality between socially and economically deprived groups has significant implications as they simultaneously suffer from multiple axes of deprivation which ultimately aggravate and intensify the distributional inequalities [19,20]. For example, it is crucial for program targeting to understand whether the service uptake by mothers (and children) from poor scheduled castes households vary from those belonging to rich scheduled castes households. Therefore, we investigate service uptake across these mutually exclusive intersectional groups defined by caste categories, household wealth and maternal education levels.

The second objective of this study was to understand the continuum in utilization of ICDS services among mothers and children in India. Continuum of care has now become a rallying call to escalate reduction in maternal and child mortality via providing a cohesive set of priority interventions throughout the lifecycle [21]. To attain rapid reductions in undernutrition, it is critical to ensure the continuum in service uptake starting from mothers (during pregnancy and breastfeeding) to child [21,22]. This study, therefore, for the first time, systematically examines the continuum—i.e., by both mothers (during pregnancy and breastfeeding) and children—in utilization of services under ICDS. Continuum in service utilization was assessed by evaluating whether mother–child pairs had received services at all three points, i.e., during pregnancy, during breastfeeding and after childbirth (up to six years). More specifically, our analysis aims to test whether those mothers who received services during pregnancy are also more likely to utilize ICDS services for their children?

## 2. Data and Methods

### 2.1. Survey Data and Study Population

The study uses data from the recent round of the National Family Health Survey (NFHS) conducted in 2015–2016. Based on Census 2011 sampling frame, the NFHS 2015–16 allows estimations on all available indicators for each of the states and union territories by rural and urban areas separately. NFHS used a two-stage stratified random sampling frame. The villages (for rural areas) and Census Enumeration Blocks (for urban areas) served as primary sampling units. In the second stage, households were selected for survey from each cluster/village/block on the basis of probability systematic sampling. The NFHS 2015–16 provides individual level information on birth history of mother–child pairs. After excluding the information on mothers who have children older than 6 years and those with missing information on child’s age, a final analytic sample of 295,646 mother–children pairs (children aged 0–6 years) with complete birth history was used for the primary analysis. It is worth mentioning here that we have considered separate pairs for those mothers with more than one child.

### 2.2. Outcomes

The binary outcome variables (yes = 1/no = 0) for the primary analysis were: (a) Whether the mother received ICDS benefits (any) during pregnancy? (survey question: *When you were pregnant with (Name of Child), did you receive any benefits from ICDS center*; (b) Whether the mother received ICDS benefits (any) while breastfeeding?) (survey question: *When you were breastfeeding (name of child), did you receive any benefits from ICDS center?*) and (c) whether the child below age of 6 years received ICDs benefits (any)? (survey question: *During the last 12 months, has (name of child) received any benefits from ICS?*). For a comprehensive understanding, we also analyzed information on specific ICDS services for both mothers as well as children. At this point it is important to mention here that benefits offered under ICDS vary across beneficiaries (i.e., mother and child). For mothers (during pregnancy and breastfeeding), we analyzed information on three specific benefits under ICDS viz. supplementary food, health checkups and health and nutrition education (HNE). Children-specific benefits includes supplementary food, health checkups, immunization and early childcare (preschool). It is worth noting here that benefits to children related to supplementary food includes both take home ration (THR) and meals at AWCs.

### 2.3. Primary Predictors

To operationalize intersectionality between socially and economically deprived groups, we constructed two categorical variables by cross classifying the following variables. At the household level, wealth index was taken as the proxy indicator for household’s income. In the NFHS, wealth index—categorized into quintiles—was created by principal component analyses on household assets and wealth characteristics for rural and urban areas separately [6]. Further, households were categorized into four social groups—scheduled castes (SCs), scheduled tribes (STs), other backward classes (OBCs) and others. Maternal education was categorized into four groups as mothers with no education, primary, secondary and higher education. The first intersectional variable was constructed by cross-classifying social groups and household wealth (20 mutually exclusive categories) and the second variable was constructed by cross-classifying maternal education and household wealth (mutually exclusive categories). The sample size of mother–child pairs in each of the intersectional category is presented in Table S1. It is important to note here that a low correlation was observed between social groups and wealth quintiles. Religion of the household head was classified into Hindu, Muslim and others.

### 2.4. Statistical Analysis

#### 2.4.1. Descriptive Analysis

All the primary statistical analyses were performed separately for rural and urban areas. The descriptive estimates regarding prevalence (in percent) of service utilization by mothers and children are presented for urban versus rural areas, by state and also by two-way cross tabulations for demographic and socioeconomic groups. In addition to this, the distribution of mother–child pairs by service utilization at different points (i.e., utilization during pregnancy, utilization while breastfeeding and utilization by child) is presented via pie graphs. For this, sampled pairs were classified into eight mutually exclusive groups as: (a) utilization by mothers during pregnancy, breastfeeding and by child; (b) utilization by mothers during pregnancy and while breastfeeding; (c) utilization by mothers during pregnancy and by child; (d) utilization by mothers while breastfeeding and by child; (e) utilization by mothers during pregnancy only; (f) utilization by mothers while breastfeeding only; (g) utilization by child only; and (h) no utilization.

#### 2.4.2. Econometric Analysis

To understand how service uptake by mothers (during pregnancy) is associated with household’s socioeconomic correlates, we fit a series of multilevel logistic regression models with random effects for village/block (level 2), district (level 3) and state (level 4). It is worth mentioning here that to understand the patterning across socioeconomic intersectional groups, we considered service uptake by mothers (during pregnancy) as an outcome variable (and not during breastfeeding and by child) in regression analysis because pregnancy is the entry point at which mothers start receiving ICDS benefits (or start visiting AWCs). In the first model, the categorical variable based on cross-classification of social groups and wealth quintiles was estimated adjusting for mother’s age, education and religion. The second model assessed the pattern in service utilization by intersectional groups based on mother’s education and wealth quintiles, adjusting for mother’s age, social group and religion. The estimates from regression analysis are presented in the form of Odds Ratios (ORs) along with respective 95% confidence intervals (CIs).

We further estimated discriminatory accuracy to identify the concordance between service uptake (any) by mothers during pregnancy and by child. More specifically, we estimated the area under receiver operating characteristic curve (AUC) that quantifies the accuracy of using individual-level information alone for identifying those with the outcome [23]. The AUC was based on post-estimation from logistic regression taking benefits received by mother during pregnancy as outcome variable; and service utilization during breastfeeding (model 1) and by child (model 2) as explanatory variable in separate models. The rationale behind taking service uptake during pregnancy as an outcome was due to the fact that at the time of pregnancy only, mothers comes into contact with services under ICDS. The regression models were adjusted for child’s age and sex, mother’s age and education, household’s wealth quintiles, social group and religion. The AUC plots the association between true positive fraction (TFP or sensitivity) on the y axis vis-à-vis false positive fraction (FPP or 1-specifity) on the x axis and ranges between 0.5 (i.e., no predictive power) and 1 (i.e., perfect discrimination). All analyses were performed in statistical software, Stata 15 [24] taking sampling weights prescribed by NFHS 2016.

## 3. Results

The service utilization (any service) by mothers during pregnancy was about 20 percentage points higher for rural areas (60.5 percent; 95% CI: 60.3; 30.7) than urban areas (38.8 percent; 95% CI: 38.4; 39.1) (Table 1). At the all-India level, about 54.3 percent (95% CI: 54.1; 54.5) of mothers were reported to utilize services during pregnancy. Even while breastfeeding, a significant gap in utilization pattern was observed between rural (55.1 percent; 95% CI: 54.9; 55.3) and urban (35.6 percent; 95% CI: 35.2; 35.9) setting with 49.6 percent (95% CI: 49.4; 49.8) prevalence for all of India. Similar pattern emerged in case of any benefits received by children (under six years) as well. For instance, about 59.6 percent (95% CI: 59.3; 59.7) of children from rural areas received any ICDS benefit, whereas about 40.2 percent (95% CI; 39.8; 40.1) received them in urban areas. About 54.1 percent (95% CI: 53.8; 54.2) of children received benefits from services under ICDS at the India level. This rural-urban gap was also evident across all types of services provided under ICDS for mothers (during pregnancy and breastfeeding) as well as children (Tables S2–S5). Among all the broad services under ICDS, service uptake of supplementary food was the highest for mothers during pregnancy both in rural (57.4 percent; 95% CI: 57.2; 57.6) and urban households (36.4 percent; 95% CI: 35.9; 36.7). On the contrary, the uptake of health and nutrition education was the lowest for mothers. For example, in rural areas, only 41.9 percent (95% CI: 41.7; 42.1) of mothers during pregnancy have received health and nutrition education under ICDS. In addition, only 42.4 percent (95% CI: 42.1; 42.7) of children in rural areas and 28.2 percent (95% CI: 27.7; 28.6) in urban areas received early childcare (preschool). Only 38.3 percent (95% CI: 38.1; 38.5) of children below six years were reported to receive early childcare under ICDS at India level.

**Figure 1:**
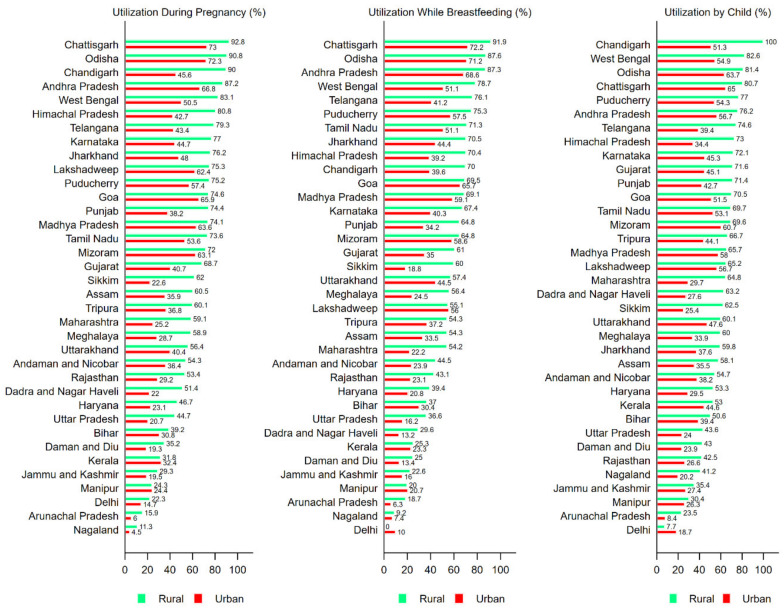
State- wise utilization of ICDS services (any) by mothers (during pregnancy and while breastfeeding) and children under six years, NFHS, 2016.

Across states, Chhattisgarh had the highest percentage of mothers receiving ICDS benefits during pregnancy both in rural (92.8 percent) as well as urban areas (73.8 percent) (Figure 1). The lowest utilization was in Nagaland (rural: 11.3 percent; urban 4.5 percent) followed by Arunanchal Pradesh (rural: 15.9 percent; urban 6.0 percent). The service utilization by children was highest for Chandigarh (rural: 100 percent; urban 51.3 percent) and lowest in Arunachal Pradesh (rural: 8.4 percent; urban 7.7 percent). Importantly, service utilization by mothers in undernutrition burdened states such as Uttar Pradesh (rural: 44.7 percent; urban 20.7 percent) and Bihar (rural: 39.2 percent; urban 30.8 percent) was very low. This calls for attention as these states are also grappling with other development issues such as high poverty and illiteracy rates. Similar patterns were observed across states foe specific services under ICDS (Tables S6–S9)

Across social groups, mothers from SC households had significantly higher utilization of ICDS services (any) during pregnancy and breastfeeding, in both rural and urban areas (Table 2). At overall national level also, about 60.9 percent (95% CI: 60.5; 61.3) of SC mothers received ICDS benefit. Compared to the general group (54.6 percent; 95% CI: 54.0; 55.01), the uptake for ICDS benefits (any) during pregnancy was about 10 percentage points higher for SC households (65.5 percent; 95% CI: 54.0; 55.1) in rural settings. This pattern was observed to be similar for country estimates as well. However, this gap was relatively higher (19 percentage points) for urban areas. In addition, the frequency of service uptake among ST was comparable to mothers from SC. A similar utilization pattern for ICDS benefits (any) was observed among nursing mothers across social groups. Among children, the service utilization was higher for SC (63.4 percent; 95% CI: 62.9; 63.8) and ST (66.1 percent; 95% CI: 65.6; 66.4) in rural areas. A similar gap was observed in urban children as well.

In rural areas, service uptake was relatively higher among mothers from middle income groups (Table 2). For instance, 60.8 percent (95% CI: 60.3; 61.2), 65.2 percent (95% CI: 64.7; 65.6) and 65.7 percent (95% CI: 65.2; 66.1) of mothers during pregnancy from second, third and fourth wealth quintiles received any ICDS benefit, respectively. At India level also, services received from ICDS were reported to be higher for mothers from middle-class households. On the contrary, estimates for urban areas revealed a clear socioeconomic gradient in service utilization with higher utilization among mothers (and children) from lower income households. For example, during pregnancy, 49.8 percent (95% CI: 49.1; 50.5) of mothers from the lowest wealth quintile received any ICDS benefit against just 19.8 percent of those from the highest wealth quintile.

Table 3 presents percentage distribution of mother–child pairs by utilization at different points of time (i.e., during pregnancy, while breastfeeding and by their children). In rural areas, about 42.4 percent of mothers had received benefits at all points, while more than one fourth had not received services at any point. The proportion of mothers utilizing services only at one point was very low (i.e., 3.6 percent during pregnancy, 8.6 percent while breastfeeding only and just 1.5 percent who availed services only for child). Similar distributional pattern was observed in urban areas with more than half of mothers (50.3 percent) receiving no benefits at all and about 26.2 percent having received services at all three points. About 7.7 percent of mothers in urban areas have received benefits only during breastfeeding and only 2.8 percent have received only during pregnancy. It is important to note that, in both rural as well as urban areas, a considerable proportion of mothers have not received any benefits under ICDS at all. At the all-India level, about 37.9 percent of mother and children were reported to receive benefits at all three points and about one third of the pairs did not avail any services at any point.

**Figure 2:**
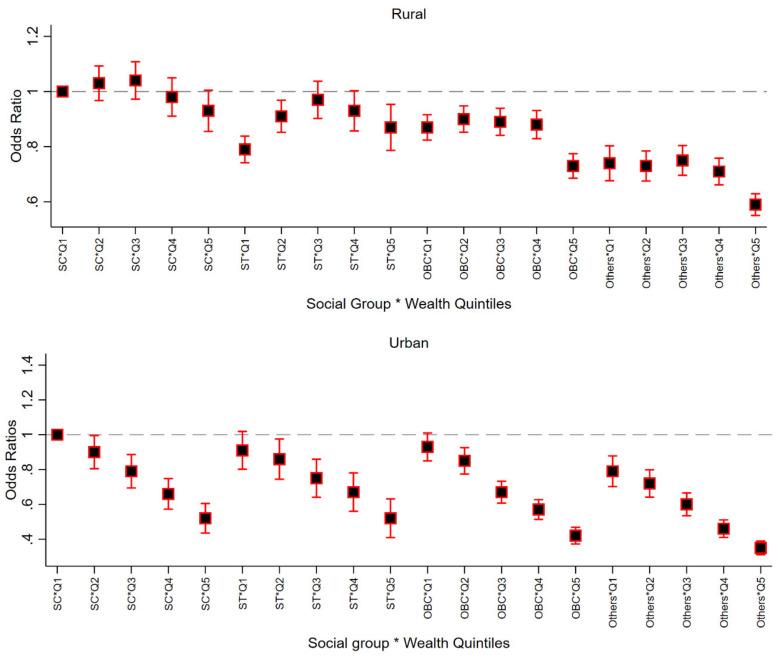
Adjusted odds ratios of service utilization (any) by caste–wealth categories of mothers, stratified by rural-urban location, NFHS, 2016; Note: Odds ratios estimated from a four level (state, district, cluster and individual) logistic regression adjusted for child’s sex, mothers’ age and mother’s education. Q1: lowest wealth quintile; Q2: second wealth quintile; Q3: third wealth quintile; Q4: fourth wealth quintile; Q5: highest wealth quintile; SC: schedule castes; ST: schedule tribes; OBC: other backward class.

Results of multilevel logistic regression models including the cross-classification of social groups and wealth quintiles in, show that compared to SC mothers from lowest wealth quintiles, the odds of service uptake were less than 60 percent for those from upper caste and highest income group (Figure 2). Surprisingly, in rural areas, ST mothers from the poorest quintile (OR: 0.79; 95% CI: 0.74; 0.84) were less likely to utilize ICDS services during pregnancy. In urban areas, a clear gradient in ORs can be observed across both social groups as well as wealth quintiles. For instance, the value of ORs for SC and ST mothers from the highest wealth quintile was 0.52 (95% CI: 0.42; 0.59) and 0.49 (95% CI: 0.40; 0.61), respectively. Similar patterns across intersectional groups were observed for service utilization by mothers while breastfeeding (Table S12).

**Figure 3:**
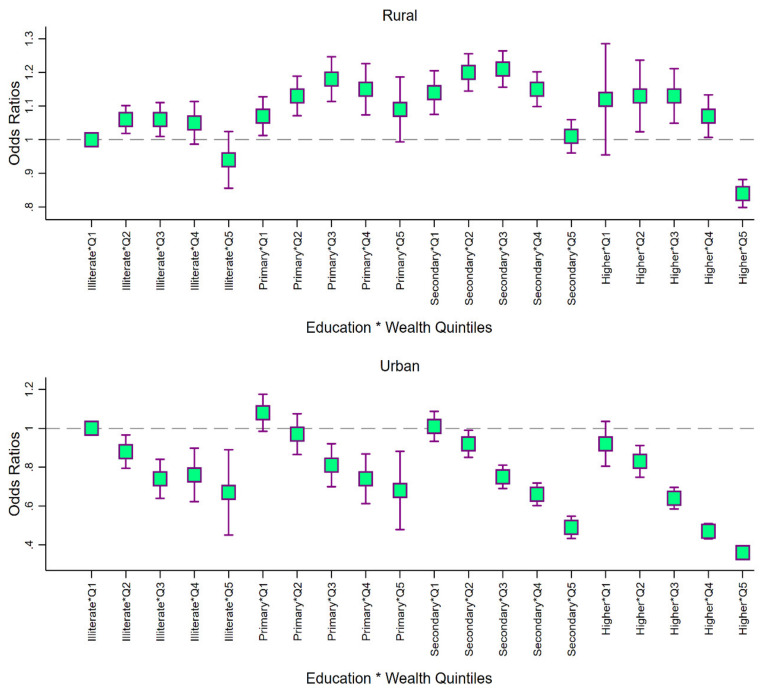
Adjusted odds ratios of service utilization (any) by education–wealth categories of mothers (during pregnancy), stratified by rural-urban location, NFHS, 2016. Note: Odds ratios estimated from a four level (state, district, cluster and individual) logistic regression adjusted for gender, social group and mothers’ age. Q1: lowest wealth quintile; Q2: second wealth quintile; Q3: third wealth quintile; Q4: fourth wealth quintile; Q5: highest wealth quintile.

We also estimated the odds of service utilization by mothers (during pregnancy) across education and income background (Figure 3). In rural setting, compared to illiterate and poorest mothers, the likelihood of receiving benefits was higher for those who had primary (OR: 1.07; 95% CI: 1.01; 1.12) and secondary education (OR: 1.15; 95% CI: 1.08; 1.20) despite being from poorest quintile. Illiterate mothers from the richest quintile had comparable odds of receiving services as those from the poorest quintile. Here also, a clear pro-poor utilization pattern was evident in urban areas with a higher probability of uptake among the less educated and poorer mothers. Importantly, none of the groups had lower odds of utilization than the highly educated mothers from richest households. In case of service uptake by mothers during breastfeeding also, the patterning across educational groups and wealth quintiles were observed to be similar (Table S13).

**Figure 4:**
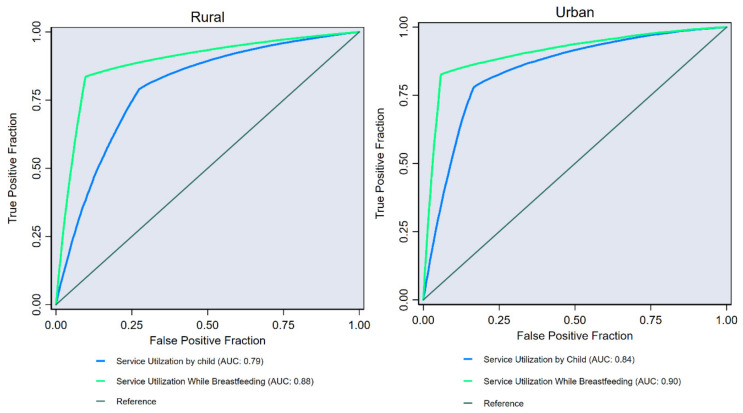
Areas under the receiver operating characteristic curve for ICDS service utilization by mother adjusted for ICDS service utilization by child and breastfeeding, India, NFHS, 2016.

To further confirm the concordance between service utilization at different points (i.e., during pregnancy, while breastfeeding and by children) we also AUC. Discriminatory analysis using AUC based on logistic regression estimates adjusted for age, sex, household wealth quintiles, religion and social group (Figure 4) showed high AUC values for both rural (AUCs < 0.88) and urban (AUCs < 0.90) areas. This indicates a high concordance between service utilization by mothers (during pregnancy) and by child. In other words, the high values of AUCs implies that likelihood of continuum in service utilization is higher if mothers have started receiving benefits during pregnancy. All the estimates are observed to be statistically significant (Table S14).

## 4. Discussion

The salient findings from this study are as follows: First, service uptake (both among mothers and children) in rural areas was noticeably higher than those in urban areas. Nevertheless, the coverage and service utilization in rural areas still needs to be improved. In fact, despite high burden of health and nutritional deprivation, service uptake in populous states like Uttar Pradesh and Bihar was extremely low. Second, among all the benefits offered under ICDS, services related to health and nutrition and preschool were relatively lesser utilized. Third, while service utilization in rural areas was higher among middle-class households, econometric analysis reveals a clear pro-poor pattern in urban households. Finally, a high concordance was observed between service utilization by mothers during pregnancy and utilization by their children. This clearly implied that likelihoods of children receiving benefits were higher when mothers received benefits during pregnancy.

This study was, however, not free from certain limitations. First, NFHS does not provide information on all the segregation of services under ICDs umbrella such as take-home ration (THR) for children aged 0–35 months and meals served at AWCs for children aged 36–72 months. However, data on all categories were available, which was sufficient to make inferences regarding utilization pattern. A relevant concern was lack of information on reasons for not opting the services which is necessary to explore possible pathways to increase service coverage. Another important limitation related to the cross-sectional nature of the NFHS was that it is not possible to infer causality between outcome and observed correlates. Absence of information on availability in terms of geographical access as well as service providers and quality are also important data limitations to be concerned.

Our findings on service uptake correspond with the estimates from existing studies based on previous NFHS rounds [25,26]. While a recent study by Chakrabarty et al. (2019) have estimated the ICDS coverage for India, but it was based on partial sample of children under five years only which do not cover some of the important component of ICDs designed for children aged 36–72 months [27]. For instance, the food supplementation strategy for children between 36–72 months is different from those between 0–36 months. Unlike younger group (0–36 months) who can avail the facility of take-home ration (THR), older children between 36–72 months are provided with cooked meals at site of AWC only.

At the state level, it is disconcerting to observe persistent substandard program coverage in most populous states such as Uttar Pradesh and Bihar, where health and nutrition outcomes are reported to be the worst. This may be primarily due to: (a) regressive distribution of program funds across states; and (b) poor governance which ultimately results in unspent program funds [10]. In the same vein, it is important to note that service utilization in Chhattisgarh—despite of having high levels of child-undernutrition—was the highest among both mothers as well as children. In addition, the success of state specific programs like the Noon Meal Program and the Tamil Nadu Integrated Nutrition Project in Tamil Nadu suggests that strong political will and commitment can reduce maternal and child malnutrition [28]. Underperforming states can perhaps benefit from some policy insights from better performing states like Tamil Nadu and Chhattisgarh.

Although service utilization in rural areas was relatively higher than urban, a large proportion of rural mothers and children still did not receive ICDS benefits. In this regard, studies have raised several concerns regarding program design and implementation [29,30,31,32]. For instance, given that beneficiaries are pregnant mothers and young children, geographical accessibility to AWCs is an important factor, particularly in rural settings where adequate transport and conveyance facilities are lacking. In this regard, a region-specific study on Wardha district has asserted that only 42.2 percent of households have an AWC within the range of one kilometer [33]. Therefore, it is important at program level to ensure appropriate placement of AWCs at an accessible distance from villages/blocks. In fact, studies have also argued that ICDS programs should be outreach based rather than center-based [34]. Further, sociocultural and religious beliefs at community level could be potent bottlenecks hindering service utilization among socially marginalized groups in rural areas. In addition to this, community level events to promote the awareness regarding ICDS benefits can potentially encourage households to avail facilities.

Further, service uptake for health and nutrition education (HNE) by mothers was lowest across all services. Here also, geographical distance from AWCs to village/blocks may be instrumental as AWWs have to provide health and nutrition education to pregnant women via home visits. In addition to this, AWWs are also expected to liaison with other frontline workers from health department (including immunization and referral services). Further, AWWs also have to provide ancillary services like IFA supplementation to pregnant mothers and deworming doses to children. This appears to be plethora of responsibilities for a single AWW at village level which perhaps makes it difficult for them to cater all the beneficiaries. More important, considering the nature and amount of duties assigned, AWWs are also observed to be inadequately trained and supervised [35]. In addition, too much reliance on food supplementation leads to a relative neglect of other effective services like pre-schooling and informal nutrition education [26]. The coverage of early childcare (i.e., preschool) for children was very low potentially aggravating the concerns regarding future human capital formation. However, POSHAN *Abhiyaan* deserves appreciation for escalating the community participation at village level, which is also crucial to increase the uptake for education related services [36,37].

We also found that service utilization was extremely low among mothers and children from socially and economically affluent background, both in rural as well as urban areas. It raises several questions regarding quality of health care system in India and further opens up great scope to understand the reasons behind not opting for services by urban middle and lower middle class. In addition, a few studies have also emphasized on a trust factor among public which plays a greater role in influencing the utilization pattern [33,38]. For example, a state-specific study based on data from Madhya Pradesh has found that AWCs which are working from more than 10 years can escalate utilization coverage [38]. A perception-based study has reported that about 75 percent of women does not feel satisfied about the quality of services under ICDS [39]. Besides infrastructural bottlenecks such as lack of space in AWCs, poor quality of food and poor diversity in food supplementation, studies have also emphasized on considering gender-related factors such as working status of mothers, child marriage for escalating service uptake in urban areas like Delhi [40,41,42] 

Contrary to expectations, the service uptake among poor and tribal (Scheduled Tribes) community was not high. Few other studies in this regard have also raises concerns pertaining to lower coverage of healthcare services as well as poor health outcomes among tribal population in India [43,44]. One possible explanation may be that AWCs are geographically inaccessible from tribal locations. Clearly, we can expect dividends from new initiatives only after ensuring program outreach to remote and tribal locations. The patterning in utilization at the intersection of caste groups and wealth revealed that caste categories matter much more than wealth in rural areas, while wealth mattered more than caste in urban areas. The impact of education beyond wealth was also highlighted. Previous studies have also highlighted the role of basic education and awareness in increasing the service uptake [45]. In addition, children with mothers who are exposed to mass media via television or Internet are found to have higher likelihood of availing services under ICDS [46]. Further a positive association has been observed between maternal education and availing preschool services for children under ICDS [47,48]. This clearly highlights the critical role of promoting female education and awareness even among urban poor. In addition to supply-side factors, demand-side features such as the perception of the scheme (a “program for the poor”?) and the expectations (longer hours? easy admission to primary schools?) need to be examined as well. These results need more nuanced inquiries into how decisions regarding utilization are made, and by whom, in different contexts and intersections.

Another important aspect to reduce child-undernutrition and mortality is continuum in maternal and childcare (i.e., from adolescence, pregnancy, childbirth and the postnatal period) [22,47]. In this regard, we observed a strong concordance between service utilization by mothers (during pregnancy and breastfeeding) and by their children. This implies a higher likelihood of service uptake by those children where mother starts receiving benefits during early pregnancy inferring continuum in service uptake. Several international and national public health researchers have in fact suggested a comprehensive set of strategies promoted the continuum in Reproductive, Maternal, New-born and Child healthcare (RMNCH) [20,21]. An important component of these strategies was to link efforts from adolescence (pre-pregnancy) to childbirth, postnatal period and childcare which was termed as *time* dimension. However, due to lack of availability of longitudinal data on maternal and child health, previous study has used cross-sectional information from NFHS 2005–06 to understand the intricacies in designing continuum in RMNCH in India at district level [49]. In this regard, studies have suggested to effectively track the progress of every prospective mother and their children in India by converging the ground-level actions of frontline health workers [49,50]. Given the wide range of services provided, ICDS can effectively ensure the continuum in maternal and childcare under single umbrella.

Using technology to avoid record duplication while effectively tracking pregnant women and their offspring, even if they move between marital and natal homes (like in PM Matritva Vandana Yojana), could lead to higher utilization rates. Addressing the drivers of these utilization patterns are critical in order to increase service uptake and achieve the nutrition targets under the POSHAN *Abhiyaan.*

## 5. Conclusions

This study examines the socioeconomic patterning in service utilization of ICDS in India on several dimensions, including continuum in maternal and childcare. The evidence shows an immense scope of improving service utilization among urban middle and lower middle-class households. This perhaps require quality improvements in the services other than food supplementation, such as health and nutrition education and early childcare (preschool). To ensure overall development of children, it is crucial to escalate the uptake of education and early childcare (preschool) related services both in rural as well as urban areas. While food supplementation is identified as an essential service under ICDS, but for holistic development of children—especially for human capital formation—education related services merits an exclusive policy focus. In the same vein, it is also critical that AWWs are provided with adequate training for their skill development. The findings from econometric analysis suggest that targeting women at the time of early pregnancy can potentially increase the likelihood of continuity in service uptake. Finally, enhancing data availability on certain service-related aspects such as, reasons for not availing ICDS benefits, education and skill of AWWs can offer valuable programmatic insights. Further research on qualitative aspects of service delivery under ICDS assumes policy salience.

## Figures and Tables

**Table 1:** Utilization of ICDS services by mothers (during pregnancy and while breastfeeding) and children under six years, India, NFHS, 2016.

Service Utilization	Rural		Urban		India	
	Utilization (%)	95% CI	Utilization (%)	95% CI	Utilization (%)	95% CI
**Mothers During Pregnancy**						
Any Service	60.5	[60.3; 60.7]	38.8	[38.4; 39.1]	54.3	[54.1; 54.5]
Supplementary Food	57.4	[57.2; 57.6]	36.4	[35.9; 36.7]	51.4	[51.3; 51.6]
Health Checkup	47.3	[47.1; 47.9]	31.6	[31.2; 31.9]	42.8	[42.6; 43.0]
Health and Nutrition Education	41.9	[41.7; 42.1]	29.7	[29.4; 30.1]	38.5	[38.3; 38.6]
**Mothers While Breastfeeding**						
Any Service	55.1	[54.9; 55.3]	35.6	[35.2; 35.9]	49.6	[49.4; 49.8]
Supplementary Food	53.1	[52.8; 53.2]	33.7	[33.3; 34.0]	47.5	[47.4; 47.7]
Health Checkup	40.5	[40.4; 40.7]	28.4	[28.1; 28.7]	37.1	[36.9; 37.2]
Health and Nutrition Education	38.0	[37.0; 38.2]	27.6	[27.2; 27.9]	35.0	[34.8; 35.2]
**By Child < 6 years**						
Any Service	59.6	[59.3; 59.7]	40.2	[39.8; 40.1]	54.1	[53.8; 54.2]
Supplementary Food	53.1	[52.9; 53.3]	35.6	[35.2; 35.9]	48.1	[47.9; 48.3]
Health Checkup	43.2	[43.0; 43.4]	30.9	[30.5; 31.2]	39.7	[39.5; 39.9]
Immunization	44.3	[44.1; 44.5]	28.6	[28.2; 28.9]	39.9	[39.7; 40.0]
Early Child Care	42.4	[42.1; 42.7]	28.2	[27.7; 28.6]	38.3	[38.1; 38.5]

**Table 2:** Utilization of ICDS services (any) by mothers (during pregnancy and while breastfeeding) and children under six years, India by Socioeconomic Background, NFHS, 2016.

Background Characteristics	During Pregnancy	95% CI	While Breastfeeding	95% CI	By Child	95% CI
**Rural**						
**Social Group**						
Schedule Caste	65.5	[65.0; 65.9]	59.7	[59.2; 60.2]	63.4	[62.9; 63.8]
Schedule Tribes	68.9	[68.6; 69.3]	64.1	[63.6; 64.5]	66.1	[65.6; 66.4]
OBC	57.7	[57.4; 58.1]	52.3	[51.9; 52.6]	56.8	[56.4; 57.1]
General	54.6	[54.0; 55.1]	49.4	[48.8; 49.9]	56.7	[56.2; 57.2]
**Wealth Quintile**						
Lowest	54.4	[53.9; 54.8]	49.8	[49.3; 50.2]	54.7	[54.2; 55.1]
Second	60.8	[60.3; 61.2]	55.3	[54.8; 55.7]	59.9	[59.5; 60.4]
Third	65.2	[64.7; 65.6]	59.3	[58.8; 59.7]	63.0	[62.5; 63.4]
Fourth	65.7	[65.2; 66.1]	60.2	[59.7; 60.7]	63.9	[63.4; 64.3]
Highest	57.8	[57.3; 58.3]	52.2	[51.7; 52.7]	57.5	[57.0; 58.0]
**Religion**						
Hindu	62.1	[61.8; 62.3]	56.7	[56.4; 56.9]	60.7	[60.4; 60.8]
Muslim	48.9	[48.4; 49.5]	44.1	[46.5; 44.6]	51.5	[50.9; 52.0]
Others	67.5	[66.9; 68.0]	61.3	[60.7; 61.9]	65.1	[64.5; 65.6]
**Urban**						
**Social Group**						
Schedule Caste	46.2	[45.3; 47.1]	42.6	[41.7; 43.5]	47.1	[46.2; 48.0]
Schedule Tribes	50.6	[49.5; 51.7]	47.1	[46.0; 48.1]	50.9	[49.8; 51.9]
OBC	41.5	[40.9; 42.0]	37.8	[37.3; 38.4]	42.4	[41.8; 42.9]
General	28.9	[28.2; 25.9]	26.4	[25.7; 27.1]	31.0	[30.3; 31.7]
**Wealth Quintile**						
Lowest	49.8	[49.1; 50.5]	46.3	[45.6; 47.0]	50.6	[49.9; 51.3]
Second	46.6	[45.7; 47.3]	42.9	[42.1; 43.7]	47.5	[46.7; 48.3]
Third	38.9	[38.0; 39.7]	35.3	[34.5; 36.1]	39.9	[39.0; 40.6]
Fourth	29.7	[28.9; 30.5]	27.2	[26.3; 27.9]	32.2	[31.4; 33.0]
Highest	19.8	[19.1; 20.6]	17.6	[16.8; 18.3]	22.2	[21.4; 23.0]
**Religion**						
Hindu	40.3	[39.8; 40.7]	37.0	[36.5; 37.4]	40.8	[40.3; 41.2]
Muslim	34.4	[33.7; 35.2]	31.8	[31.1; 32.5]	38.3	[37.5; 39.1]
Others	38.2	[37.2; 39.2]	33.2	[32.2; 34.3]	40.4	[39.2; 41.4]
**All India**						
**Social Group**						
Schedule Caste	60.9	[60.5; 61.3]	55.7	[55.2; 56.1]	59.5	[59.1; 59.9]
Schedule Tribes	66.7	[66.3; 67.0]	61.9	[61.6; 62.4]	64.2	[63.7; 64.5]
OBC	53.1	[43.8; 44.6]	48.2	[47.8; 48.5]	52.7	[52.3; 52.9]
General	44.2	[43.8; 44.6]	40.1	[39.6; 40.5]	46.3	[45.9; 43.7]
**Wealth Quintile**						
Lowest	55.5	[55.1; 55.8]	50.8	[50.5; 51.2]	55.6	[55.2; 55.9]
Second	62.3	[61.9; 62.8]	56.6	[56.2; 56.9]	61.1	[60.7; 61.4]
Third	61.3	[60.9; 61.7]	56.4	[55.9; 56.7]	59.9	[59.4; 60.2]
Fourth	52.2	[51.8; 52.6]	47.6	[47.1; 48.0]	52.3	[51.8; 52.7]
Highest	34.1	[33.6; 34.5]	30.7	[30.2; 31.1]	35.8	[35.3; 36.2]
**Religion**						
Hindu	56.4	[56.2; 56.6]	51.5	[51.3; 51.8]	55.5	[55.3; 55.7]
Muslim	43.1	[42.7; 43.6]	39.2	[38.7; 39.6]	46.2	[45.7; 46.6]
Others	58.1	[57.6; 58.7]	52.3	[51.8; 52.8]	57.2	[56.7; 57.6]

**Table 3:** Distribution (%) of mother–child pairs by utilization of ICDS services at different time points, India, NFHS, 2016.

Beneficiaries	Rural		Urban		India	
	Percent	N	Percent	N	India	N
At all three points	42.4	95,164	26.7	18,839	37.9	111,888
By mother during pregnancy and by child	6.1	13,610	3.9	2768	5.5	16,089
By mother during pregnancy and breastfeeding	8.5	19,056	5.5	3849	7.6	22,496
By mother during breastfeeding and by child	2.5	5683	2.0	1386	2.4	6992
By child only	1.5	3288	1.2	875	1.4	4133
By mother only while breastfeeding	8.6	19,396	7.7	5404	8.4	24,668
By mother only during pregnancy	3.6	8134	2.8	1960	3.4	9979
None	26.8	60,273	50.3	35,555	33.5	98,995
Total	100	224,604	100	70,636	100	295,240

Note: all three points: during pregnancy, while breastfeeding and by their child.

## Supplementary Materials

The following are available online at https://www.mdpi.com/1660-4601/17/9/3197/s1, Table S1: Sample distribution of mother-child pairs across mutually exclusive intersectional groups, India, NFHS, 2016, Table S2: Utilization (%) of ICDS service by mothers of children under six years during pregnancy by background characteristics, India, NFHS, 2016, Table S3: Utilization (%) of ICDS service by mothers of children under six years while breastfeeding by background characteristics, India, NFHS, 2016, Table S4: Utilization (%) of ICDS services by children under six years by background characteristics, rural India, NFHS, 2016, Table S5: Utilization (%) of ICDS services by children under six years by background characteristics, urban India, NFHS, 2016, Table S6: Utilization (%) of ICDS service by mothers of children under six years during pregnancy by states, India, NFHS, 2016, Table S7: Utilization (%) of ICDS service by mothers of children under six years while breastfeeding by states, India, NFHS, 2016, Table S8: Utilization (%) of ICDS services by children under six years by states, rural India, NFHS, 2016, Table S9: Utilization (%) of ICDS services by children under six years by states, urban India, NFHS, 2016, Table S10: Multilevel logistic regression estimates regarding association between utilization of ICDS services (Any) by mothers during pregnancy and socioeconomic background characteristics, India, NFHS, 2016, Table S11: Multilevel logistic regression estimates regarding association between utilization of ICDS services (Any) by mothers during pregnancy and socioeconomic background characteristics, India, NFHS, 2016, Table S12: Multilevel logistic regression estimates regarding association between utilization of ICDS services (Any) by mothers while breastfeeding and socioeconomic background characteristics and social group*wealth quintiles, India, NFHS, 2016, Table S13: Multilevel logistic regression estimates regarding association between utilization of ICDS services (any) by mothers while breastfeeding and socioeconomic background characteristics and education*wealth quintiles, India, NFHS, 2016, Table S14: Estimates for areas under the receiver operating characteristic curve for ICDS service utilization by mother adjusted for ICDS service utilization by child and breastfeeding, India, NFHS, 2016.
