# Supplementary material for: Utilization of Integrated Child Development Services in India: Programmatic Insights from National Family Health Survey, 2016
Source: Int J Environ Res Public Health. 2020 May 4;17(9):3197. doi: 10.3390/ijerph17093197 (PMC7246906; doi:10.3390/ijerph17093197)
Supplement: Supplementary file 1 [file ijerph-17-03197-s001.pdf]

International Journal of
Environmental Research and Public Health

MDPI

Supplementary Materials: Utilization of Integrated Child Development Services in India: Programmatic Insights from National Family Health Survey, 2016

Table S1. Sample distribution of mother-child pairs across mutually exclusive intersectional groups, India, NFHS, 2016.

Social Group*WQ	Rural (N)	Urban (N)	Education*WQ	Rural (N)	Urban (N)
SC*Q1	12,171	4501	Illiterate*Q1	36,122	7609
SC*Q2	10,155	2718	Illiterate*Q2	22,197	2838
SC*Q3	8653	1955	Illiterate*Q3	13,327	1237
SC*Q4	7254	1577	Illiterate*Q4	7264	654
SC*Q5	5421	1035	Illiterate*Q5	2817	183
ST*Q1	17,178	3001	Primary*Q1	8326	3625
ST*Q2	12,883	2090	Primary*Q2	9891	2095
ST*Q3	10,247	1657	Primary*Q3	8333	1212
ST*Q4	6805	1131	Primary*Q4	5644	710
ST*Q5	3689	598	Primary*Q5	2841	224
OBC*Q1	18,359	8258	Secondary*Q1	7673	6844
OBC*Q2	18,193	6882	Secondary*Q2	14,888	7400
OBC*Q3	17,133	6057	Secondary*Q3	18,688	6157
OBC*Q4	16,113	5286	Secondary*Q4	19,341	4415
OBC*Q5	15,426	3713	Secondary*Q5	15,987	2100
Others*Q1	3721	2850	Higher*Q1	901	1487
Others*Q2	5531	2942	Higher*Q2	2385	3055
Others*Q3	6369	3068	Higher*Q3	4522	4708
Others*Q4	7889	3455	Higher*Q4	7749	6106
Others*Q5	11,176	4901	Higher*Q5	15,708	7977
Total	214,366	67,675		224,604	70,636

Table S2. Utilization (%) of ICDS service by mothers of children under six years during pregnancy by background characteristics, India, NFHS, 2016.

	Rural				Urban			
	Any Services	Supplementary Food	Health Check-ups	Health & Nutrition Education	Any Services	Supplementary Food	Health Check-ups	Health & Nutrition Education
Mother's Age								
15–19	63.9	61.5	50.2	45.3	48.1	45.0	37.8	36.4
20–29	62.7	59.6	49.7	44.3	41.2	38.8	33.7	31.8
30–39	55.8	52.7	42.3	37.0	33.5	30.9	27.0	25.5
40–49	45.9	43.0	32.8	26.3	27.6	24.5	21.0	19.2
Mother's Education								
Illiterate	53.6	50.1	39.3	32.6	41.1	37.1	31.9	28.6
Primary	64.9	61.8	51.2	45.4	46.0	43.4	37.1	34.4
Secondary	67.4	64.9	55.1	50.4	44.1	41.8	36.7	34.8
Higher	60.2	57.1	48.3	44.6	36.6	35.0	30.0	28.9
College	52.6	49.4	41.7	38.4	25.2	23.2	20.2	19.7
Wealth Quintiles								
Poorest	54.4	51.1	39.5	32.5	49.9	46.9	40.1	37.0
Secondary	60.8	57.8	46.9	40.3	46.6	43.9	38.3	36.5
Middle	65.2	62.3	52.4	47.5	38.9	36.6	32.3	30.4
Fourth	65.7	63.0	53.9	49.6	29.7	27.6	24.1	23.5
Highest	57.8	54.6	46.4	42.8	19.9	17.9	15.7	14.8
Social Group								
Schedule Castes	65.5	62.7	50.8	44.9	46.2	43.5	37.9	35.3
Schedule Tribes	69.0	67.4	56.9	50.9	50.6	49.3	41.9	40.5
Other Backward Classes	57.8	54.0	44.9	39.1	41.5	38.8	33.8	31.8
General	54.6	51.6	42.1	38.5	28.9	26.6	23.2	22.1
Religion								
Hindu	62.1	59.0	49.1	43.5	40.3	37.8	33.1	31.3
Muslim	49.0	45.5	35.9	30.7	34.4	31.8	27.1	25.2
Others	67.5	66.0	50.8	48.7	38.2	36.6	30.2	29.9
All	60.5	57.5	47.4	41.9	39.4	36.6	31.5	29.9

Table S3. Utilization (%) of ICDS service by mothers of children under six years while breastfeeding by background characteristics, India, NFHS, 2016.

	Rural				Urban			
	Any Services	Supplementary Food	Health Check-ups	Health & Nutrition Education	Any Services	Supplementary Food	Health Check-ups	Health & Nutrition Education
Mother's Age								
15–19	56.9	54.5	41.4	39.1	42.1	40.7	32.1	31.1
20–29	57.2	55.2	42.6	40.2	38.0	36.1	30.5	29.6
30–39	50.7	48.6	36.1	33.5	30.4	28.5	24.0	23.3
40–49	42.5	40.3	28.0	24.4	25.0	22.8	18.7	18.3
Mother's Education								
Illiterate	48.3	46.1	32.4	29.2	36.5	34.2	27.6	26.1
Primary	58.9	56.8	43.3	40.7	43.1	41.4	33.3	32.4
Secondary	62.0	60.3	48.4	46.2	41.1	39.3	33.6	32.5
Higher	55.3	53.0	42.4	40.7	32.8	31.0	27.0	26.6
College	47.7	45.4	36.2	34.7	23.0	20.9	18.0	17.8
Wealth Quintiles								
Poorest	49.8	47.6	32.7	29.0	46.3	44.5	36.3	34.7
Secondary	55.3	53.2	39.5	36.2	42.9	40.9	34.9	34.0
Middle	59.3	57.3	45.0	43.1	35.4	33.5	28.8	27.9
Fourth	60.3	58.4	47.2	45.5	27.2	25.1	21.4	21.5
Highest	52.2	50.1	40.7	39.2	17.6	15.7	13.8	13.3
Social Group								
Schedule Castes	59.8	57.7	43.6	40.9	42.6	40.8	34.8	33.0
Schedule Tribes	64.1	62.7	49.8	46.4	47.1	45.9	37.2	37.3
Other Backward Classes	52.3	49.9	37.8	35.2	37.9	35.7	29.9	29.4
General	49.4	47.4	36.3	35.0	26.4	24.6	21.1	20.1
Religion								
Hindu	56.7	54.6	42.2	39.5	37.0	35.1	29.8	28.9
Muslim	44.1	41.8	29.9	27.5	31.8	29.6	24.3	23.8
Others	61.3	59.9	43.4	43.9	33.3	32.3	27.3	26.5
All	55.1	53.1	40.5	38.0	35.6	33.7	28.4	27.6

Table S4. Utilization (%) of ICDS services by children under six years by background characteristics, rural India, NFHS, 2016.

Mother's Age	Any Services	Supplementary Food	Health Check-up	Immunization	Early Child Care[1]	Weighed[2]
15–19	65.2	57.8	47.4	49.5	53.6	49.6
20–29	61.8	55.3	45.4	46.6	44.8	49.6
30–39	54.5	48.3	38.2	39.2	38.8	42.3
40–49	46.0	39.9	30.3	31.8	33.3	33.8
Child's Age						
0–11	61.9	53.0	43.3	50.1	0.0	43.4
12–23.	69.5	62.5	50.0	56.3	0.0	53.8
24–35	64.9	59.4	47.8	48.5	0.0	51.0
36–47	59.5	53.9	43.7	41.4	45.8	46.9
48–59	54.4	48.5	40.2	37.1	43.8	42.6
60–71	47.0	41.1	34.1	32.6	37.5	0.0
Mother's Education						
Illiterate	53.2	45.5	34.9	39.9	36.2	38.0
Primary	62.8	56.8	46.4	46.6	47.4	51.2
Secondary	65.6	60.2	50.9	49.0	48.7	55.7
Higher	60.9	54.9	45.5	44.6	42.7	50.3
College	54.7	47.7	39.2	39.3	35.4	43.5
Wealth Quintiles						
poorest	54.7	46.7	35.2	41.2	37.8	38.4
Secondary	60.0	53.3	42.6	44.5	43.2	46.3
Middle	63.0	57.1	47.4	47.3	47.1	52.0
Fourth	63.9	58.5	49.2	47.6	46.4	54.5
Highest	57.5	51.8	44.1	41.8	38.7	49.2
Social Group						
Schedule Castes	63.4	56.9	46.1	47.4	46.4	50.4
Schedule Tribes	66.1	62.4	52.4	49.5	50.6	57.8
OBC	56.8	49.1	39.5	43.7	38.2	43.5
General	56.7	50.5	41.0	39.8	40.9	45.8
Religion						
Hindu	60.7	54.0	44.2	46.1	43.1	48.7
Muslim	51.5	45.1	35.4	35.0	36.0	38.4
Others	65.1	62.7	49.1	41.9	50.2	54.5
All	59.6	53.1	43.2	44.4	42.5	47.6

1: For age 36–71 months; 2: for age 0–29 months.

Table S5. Utilization (%) of ICDS services by children under six years by background characteristics, urban India, NFHS, 2016.

Mother's Age	Any Services	Supplementary Food	Health Check-up	Immunization	Early Child Care[1]	Weighed[2]
15–19	49.3	42.8	34.9	34.1	32.4	34.7
20–29	42.4	37.8	32.9	30.5	30.0	35.7
30–39	35.1	30.8	26.6	24.5	25.3	30.1
40–49	32.4	27.8	22.0	20.4	24.2	23.5
Child's Age						
0–11	41.3	34.7	30.5	30.8	0.0	30.3
12–23.	46.1	40.6	35.1	35.3	0.0	37.3
24–35	44.8	40.7	34.6	32.1	0.0	36.9
36–47	42.1	38.1	32.9	28.4	32.6	35.0
48–59	36.0	32.6	28.3	23.6	28.0	30.1
60–71	30.7	26.7	23.8	21.7	23.5	0.0
Mother's Education						
Illiterate	42.5	35.8	30.0	31.4	29.0	33.5
Primary	47.4	42.2	35.8	33.4	35.0	40.0
Secondary	44.9	40.6	35.8	32.6	32.1	39.2
Higher	37.6	34.0	29.7	25.0	25.7	32.3
College	28.0	24.2	21.1	19.2	17.5	23.4
Wealth Quintiles						
poorest	50.6	45.0	38.3	35.9	38.4	41.9
Secondary	47.5	43.0	37.6	35.0	33.8	41.5
Middle	39.9	35.9	31.5	28.1	27.8	34.2
Fourth	32.2	27.7	24.1	22.8	19.4	26.8
Highest	22.2	18.3	16.4	14.9	14.0	18.3
Social Group						
Schedule Castes	47.1	41.9	37.0	34.3	35.2	40.7
Schedule Tribes	50.9	47.3	41.7	36.8	39.0	46.7
OBC	42.4	37.0	31.8	31.0	28.6	35.1
General	31.0	27.5	23.9	20.8	21.6	26.2
Religion						
Hindu	40.8	36.2	31.7	29.5	28.8	35.1
Muslim	38.3	33.5	28.5	26.4	26.4	30.3
Others	40.4	37.5	31.2	26.3	26.8	34.9
All	40.2	35.6	30.9	28.6	28.2	34.0

1 –For Age 36–71 months; 2 –For Age 0–29 months.

Table S6. Utilization (%) of ICDS service by mothers of children under six years during pregnancy by states, India, NFHS, 2016.

	Rural				Urban			
	Any Services	Supplementary Food	Health Check-ups	Health & Nutrition Education	Any Services	Supplementary Food	Health Check-ups	Health & Nutrition Education
State								
Andaman & Nicobar	54.3	54.0	31.6	41.6	36.4	36.4	16.9	27.5
Andhra Pradesh	87.2	86.5	74.3	81.8	66.8	66.2	53.8	60.6
Arunachal Pradesh	15.9	14.9	07.4	07.0	06.0	05.6	03.4	03.3
Assam	60.5	58.5	40.9	35.2	35.9	34.6	22.7	20.8
Bihar	39.2	34.1	24.9	18.7	30.8	25.4	19.0	15.8
Chandigarh	90.0	90.0	40.0	10.0	45.6	43.4	30.1	25.6
Chhattisgarh	92.8	92.0	84.8	81.9	73.0	71.6	64.1	65.1
Dadra and Nagar Haveli	51.4	49.9	47.3	43.0	22.0	20.0	19.3	17.6
Daman and Diu	35.2	27.4	31.8	29.1	19.3	15.4	18.6	13.8
Goa	74.6	73.5	60.3	63.9	65.9	65.0	51.3	52.9
Gujarat	68.7	66.7	61.6	56.4	40.7	37.9	35.8	33.2
Haryana	46.7	39.9	41.8	34.3	23.1	19.1	20.6	17.8
Himachal Pradesh	80.8	80.3	38.8	43.4	42.7	42.7	18.9	23.2
Jammu and Kashmir	29.3	28.3	14.9	14.9	19.5	19.1	9.7	9.8
Jharkhand	76.2	73.8	52.5	42.4	48.0	46.0	34.1	28.8
Karnataka	77.0	75.3	67.3	60.4	44.7	43.0	36.8	33.8
Kerala	31.8	30.0	19.5	22.7	32.4	30.5	18.9	21.1
Lakshadweep	75.3	75.3	4.2	13.1	62.4	62.0	25.0	28.9
Madhya Pradesh	74.1	72.6	63.6	54.7	63.6	62.0	56.1	51.3
Maharashtra	59.1	57.5	54.0	48.1	25.2	24.1	22.1	21.0
Manipur	24.3	23.9	0.8	2.2	24.4	23.6	0.6	2.3
Meghalaya	58.9	57.8	30.0	33.0	28.7	28.2	16.3	17.9
Mizoram	72.0	71.4	31.2	36.7	63.1	61.4	34.9	40.5
Nagaland	11.3	10.9	1.3	1.1	4.5	4.1	0.2	0.3
Delhi	22.3	16.7	16.7	16.7	14.7	11.7	10.7	10.9
Odisha	90.8	90.3	85.5	82.0	72.3	71.8	67.0	65.6
Puducherry	75.2	73.6	70.5	70.6	57.4	55.3	56.1	54.4
Punjab	74.4	73.3	53.3	47.8	38.2	36.9	27.2	24.9
Rajasthan	53.4	48.7	42.8	30.9	29.2	25.5	22.6	17.9
Sikkim	62.0	61.6	48.4	51.1	22.6	22.1	18.8	18.5
Tamil Nadu	73.6	71.0	69.9	67.5	53.6	51.2	50.7	48.4
Tripura	60.1	59.4	26.2	29.8	36.8	36.8	17.5	18.5
Uttar Pradesh	44.7	39.6	27.1	18.4	20.7	15.1	13.7	9.4
Uttarakhand	56.4	55.1	27.5	26.2	40.4	40.0	19.6	20.1
West Bengal	83.1	81.9	69.6	68.6	50.5	49.1	43.7	41.7
Telangana	79.3	78.5	62.1	70.8	43.4	42.9	31.4	34.8

Table S7. Utilization (%) of ICDS service by mothers of children under six years while breastfeeding by states, India, NFHS, 2016.

	Rural				Urban			
	Any Services	Supplementary Food	Health Check-ups	Health & Nutrition Education	Any Services	Supplementary Food	Health Check-ups	Health & Nutrition Education
State								
Andaman & Nicobar	44.5	44.5	27.3	36.7	23.9	23.2	16.1	20.0
Andhra Pradesh	87.3	86.5	72.7	81.5	68.6	66.7	54.0	62.5
Arunachal Pradesh	18.7	17.0	7.3	6.9	6.3	4.9	2.8	3.1
Assam	54.3	52.4	35.1	31.6	33.5	32.5	20.3	18.5
Bihar	37.0	33.4	20.4	16.6	30.4	27.0	17.0	14.4
Chandigarh	70.0	60.0	10.0	10.0	39.6	38.5	26.0	23.5
Chhattisgarh	91.9	91.0	75.6	76.0	72.2	70.7	61.0	61.9
Dadra and Nagar Haveli	29.6	28.6	27.2	23.9	13.2	12.5	12.5	11.4
Daman and Diu	25.0	19.8	21.7	20.2	13.4	11.0	12.2	12.3
Goa	69.5	69.2	54.6	58.5	65.7	65.4	54.9	54.1
Gujarat	61.0	59.4	53.3	50.1	35.0	33.0	30.7	29.1
Haryana	39.4	35.7	35.3	30.9	20.8	18.2	18.5	16.8
Himachal Pradesh	70.4	68.4	31.7	37.7	39.2	37.5	19.6	23.1
Jammu and Kashmir	22.6	21.2	12.2	12.3	16.0	14.0	7.5	7.8
Jharkhand	70.5	68.5	41.4	37.6	44.4	43.2	29.3	26.5
Karnataka	67.4	65.6	60.3	55.6	40.3	37.3	33.6	31.8
Kerala	25.3	24.0	15.3	16.9	23.3	22.1	13.6	15.0
Lakshadweep	55.1	53.1	2.1	17.3	56.0	55.0	21.7	25.2
Madhya Pradesh	69.1	67.9	54.3	48.5	59.1	57.7	51.2	48.1
Maharashtra	54.2	53.1	49.5	45.1	22.2	21.5	20.2	18.8
Manipur	20.0	19.0	0.7	1.4	20.7	20.1	0.6	1.6
Meghalaya	56.4	54.5	29.3	31.7	24.5	23.9	15.4	15.6
Mizoram	64.8	64.1	28.3	34.0	58.6	57.7	33.7	38.1
Nagaland	9.2	8.7	1.0	1.1	7.4	6.3	0.1	0.3
Delhi	---	–	–	–	10.0	9.2	6.9	7.9
Odisha	87.6	87.1	81.2	78.9	71.2	70.6	65.2	64.3
Puducherry	75.3	74.9	71.2	70.2	57.5	56.1	56.0	55.7
Punjab	64.8	63.2	44.2	42.2	34.2	33.4	24.0	22.5
Rajasthan	43.1	40.9	30.5	25.1	23.1	21.2	16.0	13.6
Sikkim	60.0	59.2	46.1	48.2	18.8	18.8	16.3	15.8
Tamil Nadu	71.3	69.6	67.8	65.9	51.1	49.5	48.3	47.0
Tripura	54.3	53.8	23.6	27.3	37.2	35.9	19.8	19.7
Uttar Pradesh	36.6	33.6	17.9	14.4	16.2	12.1	9.1	7.1
Uttarakhand	57.4	56.9	28.1	25.8	44.5	43.6	20.3	20.6
West Bengal	78.7	77.5	62.5	62.9	51.1	49.2	42.6	41.2
Telangana	76.1	74.7	58.5	66.2	41.2	39.6	27.7	31.9

Table S8. Utilization (%) of ICDS services by children under six years by states, rural India, NFHS, 2016.

State	Any Services	Supplementary Food	Health Check-up	Immunization	Early Child Care[1]	Weighed[2]
Andaman and Nicobar	54.7	53.0	39.1	16.5	41.9	55.8
Andhra Pradesh	76.2	74.7	64.3	61.4	57.1	71.7
Arunachal Pradesh	23.5	22.6	10.8	6.9	16.9	11.5
Assam	58.1	56.2	44.7	31.0	46.7	40.7
Bihar	50.6	35.3	24.3	42.2	33.8	24.2
Chandigarh	100.0	100.0	50.0	30.0	75.0	50.0
Chhattisgarh	80.7	75.2	71.1	66.0	58.7	81.5
Dadra &N Haveli	63.2	62.2	60.9	48.7	61.2	60.6
Daman and Diu	43.0	35.0	37.7	35.8	35.6	38.9
Goa	70.5	67.3	56.5	41.1	46.7	62.9
Gujarat	71.6	67.4	63.7	60.7	61.1	69.0
Haryana	53.3	46.1	44.4	46.6	38.7	48.6
Himachal Pradesh	73.0	71.0	39.0	18.7	38.2	52.3
Jammu and Kashmir	35.4	34.2	23.3	14.8	23.2	23.1
Jharkhand	59.8	55.1	35.8	46.1	32.3	45.1
Karnataka	72.1	67.8	61.5	61.5	56.9	64.4
Kerala	53.0	50.2	37.8	20.7	38.7	48.3
Lakshadweep	65.2	65.2	22.7	0.0	5.7	21.3
Madhya Pradesh	65.7	62.3	52.6	51.5	44.1	60.0
Maharashtra	64.8	62.8	58.7	51.2	61.5	61.2
Manipur	30.4	29.3	2.5	4.5	8.6	2.2
Meghalaya	60.0	59.4	38.6	21.4	40.9	46.4
Mizoram	69.6	69.2	47.6	21.6	64.1	62.6
Nagaland	41.2	40.3	4.3	6.8	6.6	5.0
Delhi	7.7	7.7	6.6	1.1	13.2	8.0
Odisha	81.4	77.9	68.7	64.2	63.9	79.1
Puducherry	77.0	73.6	71.8	58.4	64.7	75.1
Punjab	71.4	68.7	52.8	46.4	53.8	55.1
Rajasthan	42.5	35.6	28.4	33.7	22.1	31.1
Sikkim	62.5	60.7	53.9	40.9	53.5	56.2
Tamil Nadu	69.7	66.9	64.8	52.5	57.6	66.7
Tripura	66.7	64.6	43.3	31.8	59.6	52.5
Uttar Pradesh	43.6	34.0	21.6	31.8	20.9	25.3
Uttarakhand	60.1	58.0	35.4	29.0	27.3	51.6
West Bengal	82.6	81.0	72.0	47.8	75.1	78.0
Telangana	74.6	72.5	56.7	57.0	55.8	67.0
All	59.6	53.1	43.2	44.4	42.5	47.6

1: For age 36–71 months; 2: for age 0–29 months.

Table S9. Utilization (%) of ICDS services by children under six years by states, urban India, NFHS, 2016.

State	Any Services	Supplementary Food	Health Check-up	Immunization	Early Child Care[1]	Weighed[2]
Andaman and Nicobar	38.2	38.2	28.5	16.7	31.7	39.6
Andhra Pradesh	56.7	55.1	48.0	40.6	34.7	52.5
Arunachal Pradesh	8.4	7.6	4.7	3.6	5.7	4.9
Assam	35.5	34.1	26.4	17.0	25.5	25.8
Bihar	39.4	26.2	16.5	32.5	26.2	16.9
Chandigarh	51.3	44.9	32.7	28.5	38.6	38.1
Chhattisgarh	65.0	58.7	55.8	50.5	42.2	64.8
Dadra &N Haveli	27.6	24.1	22.1	15.9	23.9	22.6
Daman and Diu	23.9	20.4	19.5	17.8	17.3	19.9
Goa	51.5	49.7	41.0	31.9	33.1	51.5
Gujarat	45.1	40.8	40.2	37.5	38.2	42.5
Haryana	29.5	22.9	23.5	25.9	22.0	25.8
Himachal Pradesh	34.4	32.6	15.1	6.4	15.8	20.0
Jammu and Kashmir	27.4	25.8	18.3	10.2	18.0	19.9
Jharkhand	37.6	33.3	22.4	28.6	21.5	27.7
Karnataka	45.3	42.4	38.0	35.1	31.2	40.5
Kerala	44.6	41.2	31.0	18.2	32.2	39.4
Lakshadweep	56.7	55.4	26.6	5.9	34.2	35.6
Madhya Pradesh	58.0	54.9	49.6	45.9	38.0	55.3
Maharashtra	29.7	28.0	25.7	21.2	25.4	27.2
Manipur	26.3	25.4	3.2	3.5	10.3	2.5
Meghalaya	33.9	33.1	17.8	11.1	19.0	24.3
Mizoram	60.7	60.1	41.9	23.9	52.8	57.3
Nagaland	20.2	19.9	0.9	2.6	4.1	1.1
Delhi	18.7	14.3	12.7	13.5	11.8	13.9
Odisha	63.7	60.1	52.0	48.6	45.0	58.6
Puducherry	54.3	51.4	51.5	38.7	44.0	50.9
Punjab	42.7	39.1	28.3	25.9	27.8	30.0
Rajasthan	26.6	20.8	15.9	20.5	13.2	18.7
Sikkim	25.4	25.1	21.5	16.4	19.6	23.2
Tamil Nadu	53.1	49.6	47.2	39.1	43.4	50.0
Tripura	44.1	42.8	31.6	20.2	42.8	36.8
Uttar Pradesh	24.0	13.8	10.9	18.6	9.4	13.0
Uttarakhand	47.6	46.0	27.3	20.1	19.7	40.4
West Bengal	54.9	52.3	47.0	27.7	46.2	49.2
Telangana	39.4	37.7	29.6	28.5	23.4	33.4
All	40.2	35.6	30.9	28.6	28.2	34.0

1: For age 36–71 months; 2: for age 0–29 months.

Table S10. Multilevel logistic regression estimates regarding association between utilization of ICDS services (Any) by mothers during pregnancy and socioeconomic background characteristics, India, NFHS, 2016.

	Rural		Urban	
	OR	95% CI	OR	95% CI
Gender				
Male	1.00		1.00	
Female	0.99	[0.98; 1.01]	1.04	[1.00; 1.07]
Mother's Age (Years)				
15–19	1.00		1.00	
20–29	1.11***	[1.04; 1.18]	1.01	[0.88; 1.15]
30–39	1.05	[0.98; 1.12]	0.87*	[0.76; 0.99]
40–49	0.95	[0.88; 1.02]	0.78***	[0.66; 0.92]
Mother's Education				
Illiterate	1.00		1.00	
Primary	1.09***	[1.06; 1.12]	1.08*	[1.02; 1.16]
Secondary	1.12***	[1.09; 1.15]	1.01	[0.96; 1.07]
Higher	1.06***	[1.01; 1.10]	0.90***	[0.84; 0.96]
Religion				
Hindu	1.00		1.00	
Muslim	0.84***	[0.80; 0.87]	0.82***	[0.77; 0.86]
Other Religion	0.93	[0.88; 0.99]	1.00	[0.92; 1.09]
N	214366		67,750	

Estimates are significant at *0.10, **0.05 and ***0.01 levels. Odds ratios estimated from a four level (state, district, cluster and individual) logistic regression adjusted for gender, social group, wealth quintiles, mothers' age and mother's education.

Table S11. Multilevel logistic regression estimates regarding association between utilization of ICDS services (Any) by mothers during pregnancy and socioeconomic background characteristics, India, NFHS, 2016.

	Rural		Urban	
	OR	95% CI	OR	95% CI
Gender				
Male	1.00		1.00	
Female	1.00	[0.98; 1.01]	1.04	[1.00; 1.07]
Mother's Age (Years)				
15–19	1.00		1.00	
20–29	1.11***	[1.05; 1.18]	1.02	[0.89; 1.16]
30–39	1.05	[0.99; 1.12]	0.87	[0.76; 1.00]
40–49	0.95	[0.88; 1.02]	0.78	[0.66; 0.92]
Social Group				
Scheduled Castes	1.00		1.00	
Scheduled Tribes	1.11***	[1.05; 1.18]	1.02	[0.89; 1.16]
OBC	1.05***	[0.99; 1.12]	0.87***	[0.76; 1.00]
Other	0.95***	[0.88; 1.02]	0.78***	[0.66; 0.92]
Religion				
Hindu	1.00		1.00	
Muslim	0.84***	[0.81; 0.88]	0.83***	[0.78; 0.87]
Other Religion	0.93	[0.88; 0.99]	1.00	[0.92; 1.09]
N	224,604		70,636	

Estimates are significant at *0.10, **0.05 and ***0.01 levels. Odds ratios estimated from a four level (state, district, cluster and individual) logistic regression adjusted for gender, social group, wealth quintiles, mothers' age and mother's education.

Table S12. Multilevel logistic regression estimates regarding association between utilization of ICDS services (Any) by mothers while breastfeeding and socioeconomic background characteristics and social group*wealth quintiles, India, NFHS, 2016.

	OR	95% CI	OR	95% CI
Gender				
Male	1.00		1.00	
Female	0.99	[0.98; 1.01]	1.04	[1.01; 1.07]
Mother's Age (Years)				
15–19	1.00		1.00	
20–29	1.19	[1.13; 1.27]	1.12	[0.98; 1.28]
30–39	1.16	[1.09; 1.23]	1.00	[0.87; 1.15]
40–49	1.09	[1.01; 1.17]	0.90	[0.76; 1.07]
Mother's Education				
Illiterate	1.00		1.00	
Primary	1.09	[1.05; 1.12]	1.06	[0.99; 1.13]
Secondary	1.11	[1.08; 1.14]	1.02	[0.97; 1.08]
Higher	1.00	[0.97; 1.04]	0.81	[0.76; 0.86]
Religion				
Hindu	1.00		1.00	
Muslim	0.87	[0.83; 0.90]	0.84	[0.79; 0.89]
Other Religion	1.01	[0.95; 1.06]	1.00	[0.92; 1.09]
Social group*WQ				
SC*Q1	1.00		1.00	
SC*Q2	0.98	[0.93; 1.04]	0.86	[0.78; 0.96]
SC*Q3	0.97	[0.91; 1.03]	0.81	[0.72; 0.91]
SC*Q4	0.95	[0.89; 1.02]	0.70	[0.62; 0.80]
SC*Q5	0.91	[0.84; 0.98]	0.56	[0.48; 0.66]
ST*Q1	0.78	[0.74; 0.83]	0.90	[0.80; 1.01]
ST*Q2	0.87	[0.82; 0.93]	0.83	[0.73; 0.95]
ST*Q3	0.95	[0.89; 1.02]	0.75	[0.65; 0.87]
ST*Q4	0.92	[0.85; 0.99]	0.71	[0.60; 0.83]
ST*Q5	0.81	[0.74; 0.89]	0.49	[0.40; 0.61]
OBC*Q1	0.85	[0.80; 0.89]	0.94	[0.87; 1.03]
OBC*Q2	0.88	[0.84; 0.93]	0.87	[0.79; 0.95]
OBC*Q3	0.87	[0.82; 0.91]	0.72	[0.66; 0.79]
OBC*Q4	0.86	[0.81; 0.91]	0.60	[0.54; 0.66]
OBC*Q5	0.74	[0.69; 0.78]	0.42	[0.38; 0.47]
Others*Q1	0.72	[0.66; 0.78]	0.84	[0.75; 0.94]
Others*Q2	0.74	[0.68; 0.79]	0.77	[0.69; 0.86]
Others*Q3	0.75	[0.70; 0.80]	0.60	[0.53; 0.67]
Others*Q4	0.72	[0.67; 0.77]	0.48	[0.43; 0.53]
Others*Q5	0.61	[0.57; 0.65]	0.37	[0.33; 0.41]

Note: Odds ratios estimated from a four level (state, district, cluster and individual) logistic regression adjusted for child's sex, mothers' age and mother's education. Q1: lowest wealth quintile; Q2: second wealth quintile; Q3: third wealth quintile; Q4: fourth wealth quintile; Q5: highest wealth quintile; SC: schedule castes; ST: schedule tribes; OBC: other backward class.

Table S13. Multilevel logistic regression estimates regarding association between utilization of ICDS services (any) by mothers while breastfeeding and socioeconomic background characteristics and education*wealth quintiles, India, NFHS, 2016.

	OR	95% CI	OR	95% CI
Gender				
Male	1.00		1.00	
Female	0.99	[0.98; 1.01]	1.04	[1.01; 1.07]
Mother's Age (Years)				
15–19	1.00		1.00	
20–29	1.20	[1.13; 1.28]	1.13	[0.99; 1.29]
30–39	1.17	[1.10; 1.24]	1.01	[0.88; 1.16]
40–49	1.10	[1.02; 1.18]	0.91	[0.77; 1.07]
Social Group				
Scheduled Castes	1.00		1.00	
Scheduled Tribes	0.87	[0.84; 0.91]	0.93	[0.85; 1.02]
OBC	0.87	[0.85; 0.89]	0.92	[0.87; 0.97]
Other	0.73	[0.70; 0.75]	0.78	[0.74; 0.83]
Religion				
Hindu	1.00		1.00	
Muslim	0.87	[0.83; 0.90]	0.84	[0.79; 0.89]
Other Religion	1.00	[0.95; 1.06]	1.00	[0.92; 1.09]
Education*WQ				
Illiterate*Q1	1.00		1.00	
Illiterate*Q2	1.03	[0.99; 1.07]	0.87	[0.79; 0.96]
Illiterate*Q3	1.04	[0.99; 1.09]	0.73	[0.63; 0.83]
Illiterate*Q4	1.06	[1.00; 1.13]	0.79	[0.66; 0.95]
Illiterate*Q5	0.98	[0.90; 1.07]	0.76	[0.55; 1.05]
Primary*Q1	1.04	[0.99; 1.10]	1.05	[0.96; 1.15]
Primary*Q2	1.14	[1.09; 1.20]	0.93	[0.83; 1.03]
Primary*Q3	1.14	[1.08; 1.21]	0.80	[0.70; 0.92]
Primary*Q4	1.12	[1.05; 1.19]	0.74	[0.62; 0.88]
Primary*Q5	1.10	[1.01; 1.20]	0.83	[0.62; 1.11]
Secondary*Q1	1.13	[1.07; 1.19]	1.02	[0.95; 1.10]
Secondary*Q2	1.17	[1.12; 1.22]	0.92	[0.85; 0.99]
Secondary*Q3	1.19	[1.14; 1.25]	0.77	[0.71; 0.83]
Secondary*Q4	1.14	[1.09; 1.19]	0.69	[0.64; 0.76]
Secondary*Q5	1.01	[0.96; 1.06]	0.52	[0.46; 0.58]
Higher*Q1	1.09	[0.95; 1.26]	0.85	[0.75; 0.96]
Higher*Q2	1.12	[1.02; 1.22]	0.82	[0.74; 0.90]
Higher*Q3	1.11	[1.04; 1.19]	0.67	[0.61; 0.73]
Higher*Q4	1.06	[1.00; 1.12]	0.49	[0.45; 0.53]
Higher*Q5	0.87	[0.83; 0.91]	0.36	[0.33; 0.39]

Note: Odds ratios estimated from a four level (state, district, cluster and individual) logistic regression adjusted for child's sex, mothers' age and mother's education. Q1: lowest wealth quintile; Q2: second wealth quintile; Q3: third wealth quintile; Q4: fourth wealth quintile; Q5: highest wealth quintile.

Table S14. Estimates for areas under the receiver operating characteristic curve for ICDS service utilization by mother adjusted for ICDS service utilization by child and breastfeeding, India, NFHS, 2016.

Covariates (Outcome—Benefits During Pregnancy)	ROC Area	95% CI
Rural		
During Breastfeeding	0.889	[0.887; 0.890]
By Child	0.796	[0.794; 0.797]
Urban		
During Breastfeeding	0.906	[0.903; 0.909]
By Child	0.843	[0.840; 0.846]

Estimates are obtained from a logistic regression model adjusting for mother's age and education, child's age, household's wealth quintile, social group and religion.
